# Reconciling Pasteur and Darwin to control infectious diseases

**DOI:** 10.1371/journal.pbio.2003815

**Published:** 2018-01-18

**Authors:** Samuel Alizon, Pierre-Olivier Méthot

**Affiliations:** 1 Laboratoire MIVEGEC (UMR CNRS 5290, UR IRD 224, UM), Montpellier, France; 2 Faculté de Philosophie, Université Laval, Québec, Canada; Pennsylvania State University, United States of America

## Abstract

The continual emergence of new pathogens and the increased spread of antibiotic resistance in bacterial populations remind us that microbes are living entities that evolve at rates that impact public health interventions. Following the historical thread of the works of Pasteur and Darwin shows how reconciling clinical microbiology, ecology, and evolution can be instrumental to understanding pathology, developing new therapies, and prolonging the efficiency of existing ones.

What did the son of a tanner, born in the French region of Jura in 1822, have in common with the son of a Shropshire doctor born 13 years earlier? Both men went on to play founding roles in two major fields of biology, which are only now converging some 150 years later, with important implications for our understanding of the relationship between infectious diseases, their hosts, and the environment.

The life and contributions of these two scientists may seem radically different at first (Fig 1): while Charles Darwin worked mostly alone (despite a large network of correspondents), gathered field data to support his theories, wrote books, and did relatively few experiments, Louis Pasteur led an 'army' of research assistants who performed a wide array of experiments, wrote research articles, and typically addressed applied problems of industrial or public health interest. In addition to having different research methods, they had contrasting religious outlooks; Pasteur was known for his devout personality, while Darwin described himself as 'agnostic' late in his life. However, both researchers shared the singular ability of being able to make sense of seemingly independent observations. Both also had a profound impact on medicine during their life, without being themselves medical doctors.

**Fig 1:**
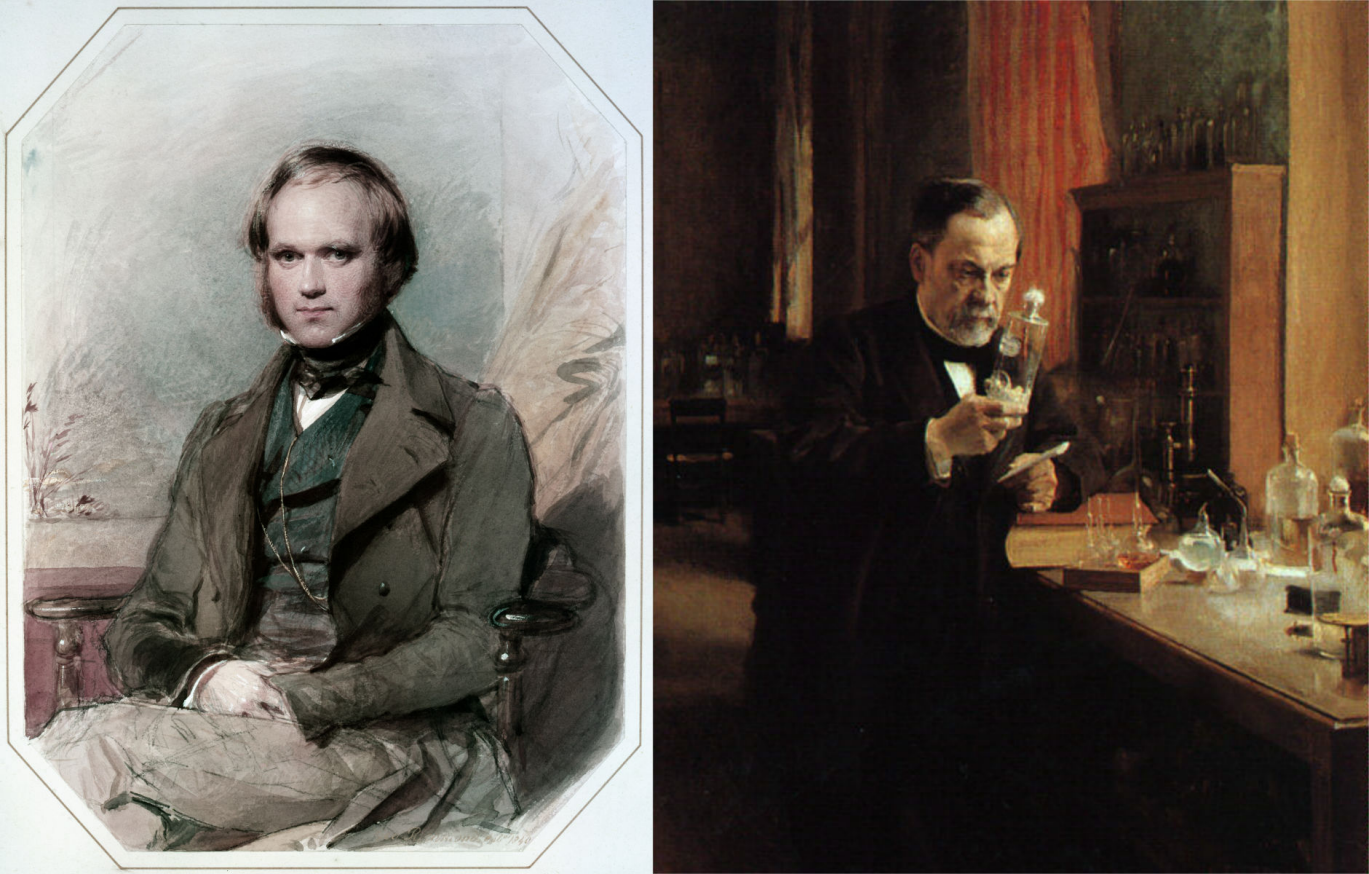
Charles Darwin the naturalist and Louis Pasteur the microbiologist. Charles Darwin's painting is from George Richmond in the late 1830s after his return from his voyage on HMS Beagle, and the painting of Louis Pasteur in his laboratory is from Albert Edelfelt in 1885 (Museé d'Orsay, Paris, France).

In a famous essay, molecular geneticist Joshua Lederberg lamented the lack of interaction between the work of the French microbiologist and the English naturalist [1]. Anecdotally, Sir James Paget mentions in his *Memoirs and Letters* (1902, p. 407) that Pasteur and Darwin both attended the International Medical Congress in London in 1881 but did not exchange words. Despite this lack of dialogue, it did not take long for microbiologists to recognize that microbes are ideal organisms to test and apply evolutionary theories [2, 3]. In fact, and contrary to a still common belief [4], not only did Darwin write about microorganisms, he even thought about how to integrate them into his theory [5]. His interest in the germ theory of disease transpired in a private correspondence with botanist Ferdinand Cohn, in which he admitted that 'if ever the origin of any infectious disease could be proved, it would be the greatest triumph to science' (p. 234 in [6]).

During Darwin's later years and not long after his death, medical doctors in Britain brought his ideas to bear on issues such as the nature and change of infectious diseases [7]. In France, the early disciples of Pasteur (the 'Pastorians'), often depicted as Lamarckian, promoted concepts of selection and variation inspired by Darwinian evolutionism [8]. Historian of science Andrew Mendelsohn even describes the laboratories of Pasteur and Koch as 'the earliest place of sustained experimental cellular-level in vitro research on phenomena understood as biological variations and evolutionary mechanisms' [9]. In retrospect, this underlying interest in variation, heredity, and (possibly) evolutionary phenomena is coherent with Pasteur's hiring of the Russian evolutionary-minded immunologist Élie Metchnikoff, according to whom 'the science of microbes has benefited from the application of the theory of evolution, and has made a fair return by supplying the Darwinian theory with a striking confirmation' [10]. Furthermore, although he did not cite the work of Darwin itself, Metchnikoff's student Charles Nicolle, director of the Pasteur Institute in Tunis for 30 years, proposed a distinctive view of the 'birth, life, and death of infectious diseases' based on the notion of 'mutation' in microorganisms and the assumption that human and animal populations can act as 'reservoirs' for the emergence of new infections [11]. The idea of harnessing ecology and evolution to control infectious diseases can therefore be traced to the work of Pasteur and Darwin, even though evolutionary biology and medical microbiology have profoundly changed, both theoretically and empirically, since then.

## Emergence of new threats

Evolutionary biology can help us understand emerging infectious diseases, as perhaps already foreseen by Pasteur [9, 12]. Drawing on his empirical work demonstrating how, after several passages in new hosts, the virulence of a microbial strain can increase for these hosts and decrease for the original host, he ventured that 'by this method new virulences and new contagions can be created'. This is how, he claimed further, 'smallpox, syphilis, plague, yellow fever appeared across the ages' [12]. These statements echoing Darwin’s theories, which at the time were upheld by atheists and by proponents of spontaneous generation [13, 14], are all the more remarkable that they came from a man who wanted to keep science and metaphysics apart [15].

This proximity between the study of disease emergence and Darwin's views has increased with the advent of microbial phylogenies. The only illustration in *On the Origin of Species* strongly resembles a phylogeny. Nowadays, thanks to DNA sequencing and computer sciences, it is commonplace to use genetic sequences from populations of microbes infecting one or several individuals to infer phylogenies. These may contain key epidemiological information if the way infectious diseases spread leaves footprints in their genomes, as postulated by the field of phylodynamics [16]. For instance, analysing avian influenza virus genomes has revealed the importance of environmental transmission in the life cycle of the virus [17]. In the case of seasonal human influenza, algorithms can integrate existing sequence data to make predictions on the most likely epitopes to emerge in the near future [18].

The 2014 to 2016 Ebola epidemics in West Africa marked a quantitative shift in sequencing with the publication of full virus genomes sampled from 78 infections within the first months of the outbreak [19]. Overall, more than one thousand complete genomes have already been analysed, thus providing us with a detailed view of the spread of the epidemics between countries [19, 20]. The early beginnings of this sequencing era could be observed during the 2009 H1N1 influenza pandemics [21]: in the case of the Ebola epidemics, phylodynamics approaches enabled researchers to go further and to infer key epidemiological parameters such as the basic reproduction ratio (*R*_0_) or infection duration [22, 23].

**Fig 2:**
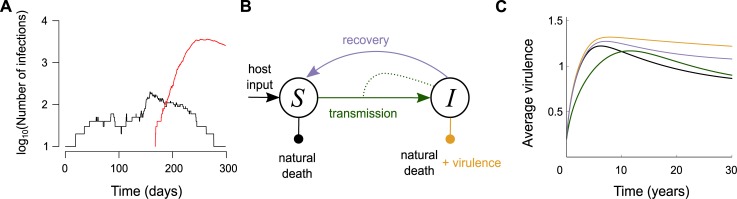
Combining the epidemiology and evolution of infectious diseases. (A) Evolutionary rescue of a parasite population via mutation, (B) representation of the SI epidemiological model, and (C) virulence evolution in response to different types of interventions. In panel A, the resident strain (in black) cannot generate a large outbreak (its *R*_0_ < 1), but it can still persist long enough for a mutation event to occur that can lead to a well-adapted mutant (in red) [26]. In panel C, the predictions are obtained using the Price equation formalism and the assumptions from Fig 2 in [30]. The colour of the curves corresponds to the arrows in panel B (black is the untreated case). Even in absence of treatment, the virulence evolves in the short term because its initial value is far from its optimal value. The virulence-blocking treatment (in yellow) leads to the highest increase virulence, whereas the treatment-blocking (in green) first favours less virulent strains. Increasing host recovery rate (in grey) also increases virulence. SI, Susceptible-Infected.

Emergence often involves adaptation to new hosts. Pasteur argued that attenuated forms of virulent parasites already exist in populations and that their 'virulence can be progressively reinforced' if the environmental conditions are adequate [9, 12]. The Chikungunya virus outbreak that occurred in La Réunion Island in 2005 to 2006 illustrates how such 'reinforcement' may occur. At the end of 2005, there was a first limited outbreak that caused a few thousand cases. In 2006, it was followed by a huge outbreak with hundreds of thousands of cases. The main reason for the size difference was that in 2006, most viruses bore a key mutation in position 226 of the E1 protein, which greatly increased the vectorial capacity of *Aedes albopictus* (tiger) mosquitoes [24], which are known for their anthropophilic behaviour. The ability to use this new vector in addition to the historical one *Ae*. *aegypti* explains the second outbreak. The evolutionary rescue framework offers an even more dynamical picture [25]: the virus population that emerged at the end of 2005 was bound to go extinct rapidly, but the evolutionary event (the substitution in position 226) allowed it to persist and generate a major outbreak. By combining epidemiology and evolution, it is possible to quantify the probability of occurrence of such a major outbreak (Fig 2A) [26]. Ten years later, a similar process might have been at work during the Ebola epidemics in West Africa [27, 28] and during the Zika epidemics in the Americas, where a mutation that enhances the virus's infectivity for *Ae*. *aegypti* was fixated into its genome [29].

## Virulence evolution

Microbial virulence is an essential concept of 19th-century bacteriological sciences [9]. Comparative pathologist Theobald Smith was among the first to formalize how virulence might evolve in the field. His theory, although more complex than usually presented [31], is that highly virulent strains would be counterselected because killing their host is detrimental to their epidemiological fitness [32]. Smith's contributions led to new ways of looking at hosts and parasites that ran parallel to the more conventional and dominant 'magic bullet' narrative in the 20th century, which postulates that we will always find a drug to selectively kill any microbe [33]. After Smith, medical scientists such as Charles Nicolle, Karl F. Meyer, Frank Macfarlane Burnet, René Dubos, or Frank Fenner promoted the study of the natural history of infectious diseases. Understanding and controlling epidemics, they argued, was not reducible to tracking pathogenic germs in their remotest 'nook and crany', to quote Robert Koch—a practice embodied by the 'microbe hunter' tradition in bacteriology [34].

Although hugely influential, Smith's claim that parasites would always become avirulent given enough time did not go unchallenged. In the early 1980s, building on important advances in evolutionary theory recognizing that selection can operate simultaneously at multiple levels [35], evolutionary epidemiologists argued that virulence—usually measured through the host's parasite-induced mortality rate—can be adaptive if trade-offs are involved [36, 37]. Some of the clearest support for the trade-off hypothesis in a human disease comes from HIV-1 [38]. HIV virulence is classically measured in the absence of treatment as the inverse of the time to AIDS. Therefore, by definition, HIV virulence decreases infection duration. Increased virulence, however, also comes with increased probability of transmission per sexual contact. The trade-off originates from these two opposite forces: milder viruses cause longer but poorly contagious infections, whereas virulent viruses cause short but contagious infections. Mechanistically, this relationship has been shown to be mediated by HIV set-point viral load, which is correlated with both virulence and transmission. Furthermore, this virus load has been shown to be at least partly controlled by the virus's genetics [39, 40]. Observed evolutionary dynamics are consistent with the existence of such a trade-off [38]. Of course, explanatory hypotheses of field data are rarely mutually exclusive, and HIV virulence could also be due to maladaptation or to other selective forces such as multiple infections. More generally, when epidemiological fitness components other than transmission rate and infection duration are considered, establishing the adaptive nature of virulence is challenging [41].

A direct epidemiological consequence of this trade-off is that population dynamics feedbacks can generate a transient advantage for virulent strains early in epidemics [30]. This short-term effect was shown in vitro by generating an outbreak in a bacterial population with a mixture of virulent and nonvirulent phages. The initial 1:1 ratio first increases to 100:1 before decreasing to 10:1 in favour of the virulent phage [42]. Field data on the spread of pathogenic bacteria in North American house finch also support this theoretical prediction [43]. This initial peak in virulence makes emerging infectious diseases even more dangerous in the short term (not to mention that the lack of immunity in the host population can amplify this increased virulence).

The trade-off also has ethical implications because any public health intervention that affects parasite transmission may impact virulence evolution (see Box 1 on vaccination). Often, what is best for an individual in terms of antiparasitic treatment may be at odds with what is best for the population, as in the case of antibiotic resistance [44]. In such a case, evolutionary biologists can help clinical microbiologists and epidemiologists because they have a tradition of studying processes at different scales [35].

**Box 1. Vaccination: When resistance meets virulence**

Pasteur’s privileged way of fighting infectious diseases was vaccination. Although this can impact the relation between a host population and its parasites, the evolutionary effects of vaccination are less predictable and often less apparent than the effects of using antimicrobial drugs [45].

One of these effects is vaccine escape, which describes infections caused by a parasite variant that has a different epitope (antigenic determinant) than the one targeted by the vaccine. This process is similar to drug resistance and has been described in the case of hepatitis B virus, *Bordetella pertussis*, and *Streptococcus penumoniae* [46].

In addition to this 'evasion' scenario, an 'escalation' scenario is possible [47] in which the parasite variant that successfully spreads among vaccinated hosts is more virulent than parasites that go extinct. From a molecular point of view, this can be caused by epitope changes (in which case there is both evasion and escalation) or by an increase in the replication rate.

Vaccines can interfere with the parasite's life cycle in several ways (Fig 2B), and evolutionary theory predicts that the mode of action impacts the nature of the fittest strain [48] (Fig 2C). This was shown experimentally using the rodent malaria parasite *Plasmodium chabaudi* in mice: a replication-blocking vaccine led to the evolution of higher levels of virulence during serial passage experiments [49]. In theory, interventions that only reduce infection virulence, thereby turning infected hosts into healthy carriers, are the most dangerous ones in the long term [48]. Recent studies have found that using virulence-blocking vaccines against Marek Disease Virus (MDV), an avian herpesvirus and a major threat to the poultry industry, support these predictions. Until recently, the evidence was mainly correlational [50], but experiments now show that vaccinating chicks can lead to the emergence of more virulent strains otherwise unable of generating any secondary case and spreading into the population [51].

Vaccination is a case in point about how bringing evolutionary ecology and clinical microbiology closer to one another could open new perspectives. Whereas the latter tends to focus on molecular and cellular interactions leading to evasion and escalation, the former studies the effects of vaccination at the host population level, thus making a number of simplifying assumptions (for instance, in classical models, a vaccine cannot act in multiple ways, e.g., decreasing both infectivity and replication, and parasites cannot have multiple traits evolve such as virulence and escape). Increased realism about the mode of action of vaccines and the evolutionary response of parasites will require a closer dialogue between distinct domains within the biomedical sciences.

## Resistance evolution

In 2000, the frequency of *S*. *pneumoniae* resistant to penicillins (J01C β-lactam antibacterials) was shown to strongly correlate with the amount of antibiotics from this class prescribed per inhabitant in European countries [52]. Although the strength of the correlation came as a surprise, the problem itself was not new. The observation of resistant bacterial strains was concomitant with the introduction of antibiotics in the mid-1940s [53]. The ensuing decades, which were characterized by large-scale use of antibiotics in medical and nonmedical contexts, facilitated the emergence of resistant and super-resistant bacteria. Warnings from Fleming in his Nobel lecture in 1945 and later from ecologically minded medical scientists such as Dubos and Burnet on the consequences of resistant microorganisms selected by the use of antibiotics remained largely unheard [53]. Metaphorically, Dubos described doctors faced with the problem of infectious diseases as 'gardeners whose work never ends' and argued that 'students of disease must always be on the lookout for new problems of infection' [54]. Ecological perspectives on disease were never absent in 20th-century biomedical science, but until the 1980s and the outbreak of the AIDS pandemic, they did not attract as much attention as the discovery of new drugs, the eradication of a particular disease like smallpox, or the identification of new pathogens did [55]. The prospect of an 'empty pipeline', the resurgence of resistance worldwide, the failure of eradication programmes, and changes in natural environment as well as greater awareness that microbes are evolutionary agents provided a wider space for 'ecological reasoning in medicine' [33].

Parasite virulence has been under scrutiny by clinical microbiologists since Pasteur [56], but it has also been a central trait for evolutionary biologists [41, 57]. Some exceptions notwithstanding [58, 59], this is not the case for antibiotic resistance. Evolutionary biology studies investigating how to best combine antibiotics [60] or alternate between them [61] are recent compared to the time since these practices have been attempted in the medical field [62]. In epidemiology, ecological theory has provided methodological insights. For instance, using a Bayesian analysis of prevalence data from Cuba, Venezuela, and Estonia, a research team has estimated the fitness cost paid by resistant strains of *Mycobacterium tuberculosis* [63]. Based on datasets from the United States and Ireland, it has also been argued that larger hospital sizes favour the spread of antibiotic resistance, one interpretation being that a network of small hospitals maximises the risk of stochastic extinctions of newly emerged resistant variants [64].

**Fig 3:**
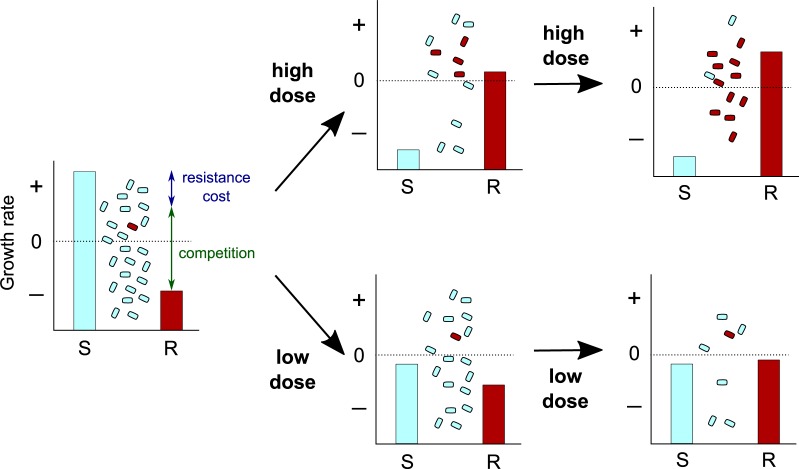
How high drug doses can lead to selection of preexisting drug-resistant mutants via 'competitive release'. Charts show the within-host growth rate of drug-susceptible (in cyan) and -resistant (in red) bacteria populations at three time points using a high (top) or a low (bottom) drug dose. Population sizes and the fraction of resistant bacteria are shown between the bars. Note that depending on the fitness landscape, there might not always exist a dose that prevents the spread of both bacterial populations (for details, see [68]). R, Resistant; S, Susceptible.

The renewed focus on the evolution of drug resistance has led evolutionary biologists to challenge Paul Ehlrich's dogma for treating infections ('Frapper for et frapper vite', or 'Hit early and hit hard' in English [65]). As illustrated in Fig 3, it may sometimes be preferable to use lower doses to contain an infection instead of attempting to eliminate it [66]. Although provocative and challenging from a clinical point of view, similar 'watch and wait' strategies are already routinely implemented to treat chronic lymphocytic leukemia [67]. Evolutionary biology can offer a conceptual framework to guide these practices because choosing the optimal strategy is sometimes counterintuitive. For instance, one of the few early mathematical models from the 1990s showed that rotating between different antibiotics at the population level selected for higher levels of resistance than treating part of the population with different antibiotics [59], thus warning against the consequences of an empirical 'trial and error' approach.

### Seeding evolutionary theories

Evolutionary biology currently has a marginal place within medicine. There is even a significant tendency to avoid the 'e-word' in the biomedical literature when referring to antimicrobial resistance [69]. Yet in the 19th century, medical sciences were as enthusiastic for Darwin’s ideas [70] as they were initially hostile to Pasteur's [71]. This support, often implicit, progressively came to a halt in the 20th century for at least two reasons. First, the intellectual proximity between evolution, eugenics, and medicine, most clearly articulated in Karl Pearson's 1912 address ('Darwinism, medical progress, and eugenics') and in George Draper's constitutional medicine [72], made scientists wary of implementing evolutionary approaches in medicine, particularly after World War II. Experimentation on human subjects in Nazi Germany revulsed public opinion worldwide and ended eugenic policies, at least in the public discourse [73]. Second, the major reform in US medical education along the lines promoted by Abraham Flexner in his 1910 influential report contributed to insulating evolutionary thinking. At the time, evolutionary biology was still largely viewed as an observational science and no longer had a place within the new configuration of medical knowledge and training organized around specialities and characterized by experimentation [74]. In addition to this ban from medical curricula, some prominent evolutionists of the Modern Synthesis such as Theodosius Dobzhansky [75] and Ernst Mayr [76] typically evinced little interest in the evolution of microorganisms.

For some medical bacteriologists in the interwar period, however, the nature of microbial species in relation to virulence and variability raised fundamental problems that were often depicted in evolutionary terms. Changes in microbial colonies (from smooth to rough, or vice-versa), as described by Fred Griffith in the 1920s, for instance, compelled bacteriologists to investigate 'heritability', 'mutation', or 'adaptation' of these traits. The study of changes in bacterial species, Griffith argued, was important for epidemiology because it 'may some day provide an explanation why a ubiquitous and apparently harmless organism may suddenly become more pathogenic for its host and of such high infectivity as to propagate an epidemic' [77]. Still, it took several more decades before evolutionary perspectives on the biology of infectious diseases reached the bedside and informed clinical medicine [78].

There is now increasing support for the teaching of evolutionary biology in medical faculties [79, 80]. When teaching medical students, however, one should call attention to the set of assumptions often made regarding ancestral lifestyles [81] or the adaptative value of certain traits or behaviours [82]. Furthermore, it should be emphasized that medicine and evolution have different ‘conceptual bases’ and are typically concerned with different problems: whereas the former focuses on restoring health at the individual level, the latter studies biological variations at the population level and how they change over time [83]. For these reasons, the greatest potential impact of evolutionary medicine is likely to come from a better integration of microbial evolution in public health and epidemiology [80]. In turn, this raises the question as to whether introducing evolutionary biology in public health departments rather than amending (dense) curriculae in medical faculties might be a more efficient strategy.

Just like evolutionary theory, public health is typically concerned with the study of populations, making it theoretically closer to clinical medicine. One of the ways this is already being implemented is through journals and learned societies. Furthermore, evolutionary ecologists who work on health-related topics should not hesitate to attend workshops and other scientific meetings organised by their national public health agencies.

At the individual level, the greatest potential impact of evolutionary theory has to do with the within-patient evolution that occurs during infection by rapidly evolving pathogens or during cancer. One difficulty is that this requires not only convincing medical doctors of the significance of selection processes at this level, but also of the usefulness of concepts and methods from population dynamics and community ecology to study cellular dynamics [84]. To this end, creating bridges between molecular biology, ecology, and evolution departments might be the most efficient way to have a concrete effect. Indeed, our historical approach highlights that these bridges should not be taken for granted. Incidentally, this brings us back to Pasteur's fight to introduce microbiology in public health and Darwin's work to bring all available biological facts into his theory. Extending their efforts should help broaden the perspective for clinical research and help improve designing treatment protocols and national guidelines.

## Mastering coevolution

Over the years, Darwin and Pasteur's fields have drifted apart due to a combination of historical, social, and epistemological factors. Evolutionary ecologists tend to focus on environmental processes and phenotypic traits at the expenses of the underlying molecular and cellular interactions, whereas clinical microbiologists work in the opposite direction (see Box 1). A more synthetic view is not only necessary to understand pathogenesis, but also to develop and enhance therapies as well as to reduce morbidity and mortality. This is also likely to raise ethical issues that cannot (and should not) be tackled by scientists alone.

The stakes are higher than not being able to use a few drugs. There is a serious risk that in the long run, policies might trigger an arms race favouring more virulent parasite strains, as witnessed for MDV in the poultry industry (see Box 1). Similar concerns apply to the use of antimicrobial peptides in clinical and agronomical practices. Because these peptides are part of our own defence measures, evolution of bacterial resistance could potentially reduce the efficacy of our immune systems [85]. More generally, there is a documented risk of coevolution between drug resistance and virulence [86]. Microbial evolution in response to public health interventions is almost unavoidable; at the same time, the diversity of the microbial population shapes the public health response because mild strains are less reported and treated than virulent ones. Actions can be undertaken, however, to ensure that the outcome of this coevolutionary process tends towards a more peaceful coexistence rather than an arms race [87].

In conclusion, there is an urgent need to switch from an eradication to a control perspective as already advocated in 1955 by René Dubos [88] or in 2000 by Joshua Lederberg [55]. We should add to the search for 'magic bullets’ the development of strategies to manage and mitigate pathogen evolution. In that sense, interventions that have a strong ecological and evolutionary dimension, such as microbiota transplantation, new ways of administrating drugs (varying doses, alternating or combining molecules), or even advances in phage therapy, could be the future of public health [89, 90]. Darwin and Pasteur did not speak to each other at the conference they attended, but it is more urgent than ever that the two fields they helped create start conversing in the 21st century.

## Abbreviations

I: Infected
R: Resistant
S: Suspectible
